# Enhancement of viral escape in HIV-1 Nef by STEP vaccination

**DOI:** 10.1097/QAD.0000000000001202

**Published:** 2016-09-28

**Authors:** Sung Yong Park, Wendy J. Mack, Ha Y. Lee

**Affiliations:** a Department of Molecular Microbiology and Immunology; b Department of Preventive Medicine, Keck School of Medicine, University of Southern California, Los Angeles, California, USA.

**Keywords:** HIV-1, Nef, vaccine, viral escape

## Abstract

Properly priming cytotoxic T-lymphocyte (CTL) responses is an important task in HIV-1 vaccination. However, the STEP trial showed no efficacy even though the vaccine elicited HIV-specific CTL responses. Our study is to investigate whether or not the STEP vaccine enhanced viral escape in infected volunteers.

**Methods:**

The signature of viral escape, the presence of multiple escape variants, could be falsely represented by the existence of multiple founder viruses. Therefore, we use a mathematical model to designate STEP study patients with infections from a single founder virus. We then conduct permutation tests on each of 9988 Gag, Pol, and Nef overlapping peptides to identify epitopes with significant differences in diversity between the vaccine and placebo groups using previously published STEP trial sequence data.

**Results:**

We identify signatures of vaccine-enhanced viral escape within HIV-1 Nef from the STEP trial. Vaccine-treated patients showed a greater level of epitope diversity in one of the immunodomiant epitopes, EVGFPVRPQVPL (Nef_65–76_), compared with placebo-treated patients (*P* = 0.0038). In the other three Nef epitopes, there is a marginally significant difference in the epitope diversity between the vaccine and placebo group (*P* < 0.1). This greater epitope diversity was neither due to any difference in infection duration nor overall *nef* gene diversity between the two groups, suggesting that the increase in viral escape was likely mediated by vaccine-induced T-cell responses.

**Conclusion:**

Viral escape in Nef is elevated preferentially in STEP vaccine-treated individuals, suggesting that vaccination primarily modulated initial CTL responses. Our observations provide important insights into improving vaccine-primed first immune control.

## Introduction

HIV-1 vaccine-design efforts have in large part centred on T-cell priming immunizations, as the pivotal role of CD8^+^ T cells in controlling viral replication has been demonstrated both in HIV-1 [1–3] and experimental SIV infections [4–6]. The STEP HIV-1 vaccine trial, a double-blind phase 2b study, did not show efficacy in prevention of HIV acquisition or reduction of early viral load, even though the vaccine construct of HIV *gag*, *pol*, and *nef* genes elicited HIV-specific CD8^+^ T-cell responses in more than 70% of the vaccinated individuals [7,8]. Although the preplanned analysis revealed no statistically significant enhancement of HIV acquisition in the vaccine group, post-hoc analyses of subgroups indicate increased HIV infection in vaccinated patients who either had prior immunity against the Ad5 vector or were uncircumcised [7,9]. The fact that the vaccine was deemed both inefficient and even harmful in certain populations necessitates more specific characterization of T-cell responses for next-generation vaccine design.

Although both the magnitude and breadth of vaccine-primed CD8^+^ T-cell responses were far from optimal for protection [10], the STEP trial did impact breakthrough viral populations as viruses infecting vaccine recipients were more likely to have epitopes different from those in the vaccine [11]. The two most plausible scenarios causing these genetic modifications by vaccination were a sieve effect and an increase in viral escapes [11]. It is possible that vaccine-elicited cytotoxic T-lymphocyte (CTL) responses created selective pressure on breakthrough viruses (sieve effect), preferentially transmitting viral strains with epitopes more different from those in the vaccine within vaccine recipients as compared with placebo recipients [11]. The viral escape scenario proposes that a strong CTL response mounted by vaccination provoked more frequent viral escapes in vaccinated individuals, resulting in greater divergence from the vaccine strain. To more rigorously understand the viral escape scenario, we aim to identify the signature of vaccine-enhanced viral escapes from the STEP trial.

Viral escape from CTL was reported to be prominent as early as 20–30 days after the acute peak of viraemia with the median rate of escape of 0.14 per day [12]. This acute escape rate is substantially greater than the chronic escape rate that was estimated as 0.04 per day [13], implying that CTL-mediated killing is more prominent during the acute than the chronic stage. One characteristic of viral escape is a significant increase in epitope diversity or epitope entropy; the transmitted form of a CTL epitope is typically depleted as early as 2 weeks after the first screening, whereas epitopes with varied escape mutations subsequently appear and coexist for more than 1 year [12,14]. This observation indicates that a robust signature of viral escape is nonzero epitope diversity as a result of the coexistence of diverse epitope sequences. Serial sequence data from experimental SIV infections have confirmed this aspect of HIV-1 escape [15].

There have been varied observations about the impact of escape on viral replication and disease progression. Several studies reported an association between rapid disease progression and escape from CTL pressure [16,17]. In contrast, early viral escape may cause a transient viral load increase but ultimately result in improved control of viraemia by more effectively inducing persistent T-cell responses targeting conserved regions of viral proteins [12]. Other studies demonstrated that CTL escape had no effect on disease progression [18,19]. Taken together, the outcome of viral escape on long-term viral control considerably varies across individuals.

We seek to identify the signature of viral escape – the presence of heterogeneous epitope sequences – from the STEP trial sequence data. The presence of diverse epitopes could be represented by the existence of multiple transmitted founder viruses. To avoid this type of false positive result, we exclude patients infected by multiple founder strains. By comparing epitope diversity only between STEP vaccine and placebo recipients whose infections originated from a single founder virus, we directly assess the effect of vaccination on early viral escape. Understanding how vaccines alter or impact viral escape is a critical step towards developing an efficacious HIV-1 vaccine.

## Methods

### Sources of STEP-trial sequence data

Out of 68 STEP study participants’ published whole genome sequence data [11], a total of 59 STEP study patients’ data were used for our analyses. All patients’ specimens were taken from United States, Canada, or Peru at the time of HIV-1 diagnosis or 1 month later. Patients 502-0006, 502-0223, 502-1368, and 502-1919 were excluded from our analysis because their number of envelope gene sequences was less than three. To probe the impact of the vaccine on early HIV-1 escapes, only recently infected individuals were included in our analysis; patients 502-0965, 502-1115, 502-1709, 502-2000, and 502-2008 were excluded because the genomic HIV incidence assay [20,21] identified these patients as chronically infected. The incidence assay genomic biomarker, the 10% quantile of Hamming distance distribution of the full envelope gene sequences, was measured to be 13, 11, 9, 8, and 12 for patients 502-0965, 502-1115, 502-1709, 502-2000, and 502-2008, respectively, suggesting a chronic infection. The HIV *gag, nef, pol,* and *env* gene sequences of the remaining 59 infected individuals (37 vaccinees and 22 placebo recipients) were subjected to further analyses.

### Estimating timing of infection and number of founder/transmitted viruses

The shifted Poisson mixture model (SPMM) [22] was used to estimate the number of founder variants and time since infection from each STEP patient's envelope gene sequences. When a recent infection originates from a single variant, the Hamming distance distribution of sequences would have only one peak at low Hamming distance region, indicating the presence of identical and/or closely related sequences. In case of multivariant transmissions, additional peaks at distances between founder sequence pairs are present. By collecting the probability distributions of the Hamming distances within viral lineages and those among lineages, we obtain a patient's Hamming distance distribution with *k* founder lineages as follows, as described in Ref. [22], 
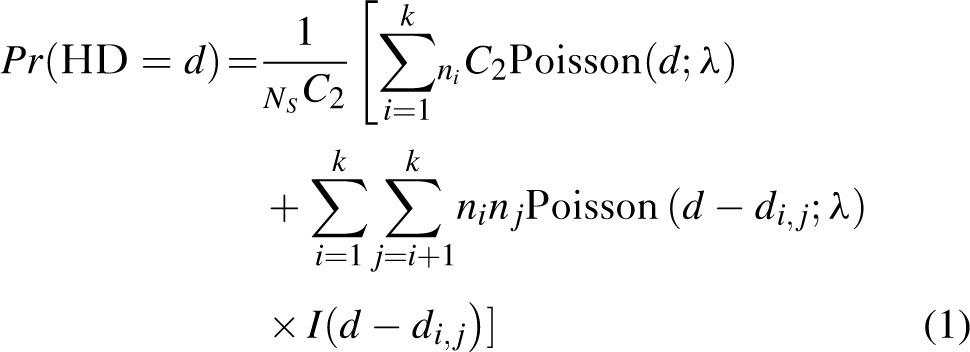


where *N*_*s*_ is the number of sampled sequences,  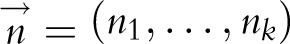 is the number of sampled descendants of each of the *k* founder strains,  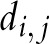 is the Hamming distance between *i* and *j* founder sequences, and  
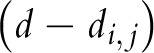
 is an indicator that distances between founder strains must be smaller than observed Hamming distances between sequence pairs. Here, the Poisson parameter λ has a linear relationship to time since infection, *t*, 
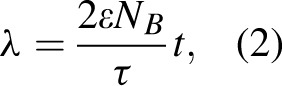


where ε is the rate of base substitution by HIV-1 reverse transcriptase, *N*_*B*_ is the number of bases in the sequence, and τis viral generation time [23,24]. Using the method of conditional maximization, the best fit of the SPMM in Eq. (1) to each STEP patient's Hamming distance distribution was obtained, providing estimates of days after infection and the number of founder variants.

As shown in Love *et al.*[22], the proper usage of the SPMM requires prescreening for recombinant sequences and the signature of APOBEC3G/F-mediated hypermutation [25]. All alignments were checked for recombination using the Recombination Detection Program version 3 [26,27] in tandem with manual inspections. All recombinants were removed prior to our SPMM analysis, as presented in Supplementary Table SI. Similarly, as shown in Table 1, hypermutation signatures were removed using the Los Alamos National Laboratory tool Hypermut (http://www.hiv.lanl.gov/content/sequence/HYPERMUT/hypermut.html).

### Epitope library construction

We constructed a library of CTL epitopes spanning the HIV Gag, Pol, and Nef proteins of the STEP vaccine construct, MRKAd5 [7,8]. The library contains all documented HIV epitopes in the Los Alamos National Laboratory HIV database (http://www.hiv.lanl.gov). In addition, potential epitopes were predicted by the software NetMHC [28,29]. We inputted a library of unique HIV Gag, Pol, and Nef amino acid sequences obtained from the STEP study patients into NetMHC. NetMHC then predicted the binding of overlapping peptides (8–14 amino acids long) in each input amino acid sequence to one or more of the 78 HLA types listed in NetMHC. The synthesis of the Los Alamos National Laboratory HIV database with NetMHC predictions generated a total of 2648 Gag, 5995 Pol, and 1345 Nef overlapping peptides.

### Permutation *t* test

We statistically examined differences in the epitope diversity between the vaccinated and placebo individuals whose infections originated from a single variant. The epitope diversity was defined as the average number of amino acid changes between all possible sequence pairs of a single patient divided by the length of the peptide. We conducted a permutation test to empirically assess the validity of the asymptotic *P* values. For each epitope, a *t* statistic was computed on the observed diversity data as follows: 
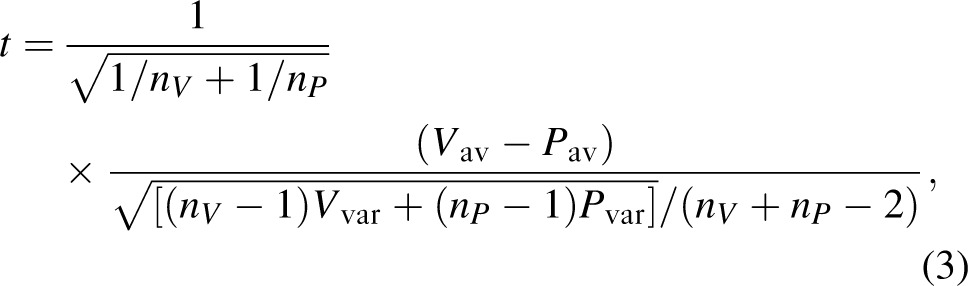


where *n*_*V*_ (*n*_*P*_) is the number of vaccine (placebo) patients,  
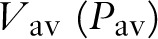
 is the average epitope diversity among vaccine (placebo) patients, and  
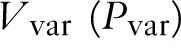
 is the variance of the epitope diversity among vaccine (placebo) patients. The treatment group was then randomly permuted across patients, holding the numbers of vaccine-treated (*n*_*V*_ = 29) and placebo-treated (*n*_*P*_ = 20) patients constant over permutations; a total of 10 000 such permutations were completed. For each permutation and epitope, a *t* statistic in Eq. (3) was computed. The empiric *P* value of each epitope was calculated as the proportion of permutations with a *t* test statistic greater than or equal to the *t* test statistic derived from the actual data. Based on this empiric *P* value, the epitopes with statistically significantly greater diversity in the vaccine-treated than the placebo-treated patients were designated as vaccine-enhanced escape epitopes.

## Results

### Identifying vaccine and placebo patients with a single-strain infection

As demonstrated in HIV-1 and SIV infection studies [14–16], early viral escapes are characterized not only by amino acid substitutions from the founder epitope sequence, but also by the presence of multiple escape variants [30]. In response to initial CD8^+^ T-cell responses, multiple mutants of the transmitted/founder epitope sequence appear and coexist throughout the early stages of infection. Nonzero epitope diversity can thus indicate viral escape. This epitope diversity proxy, however, cannot hold in infections from multiple founder viruses, in which case epitope diversity can arise from the transmission of different viral lineages. To eliminate false positive escape signals, we classified all patients’ infection to only include patients with infections from a single founder virus.

**Fig. 1:**
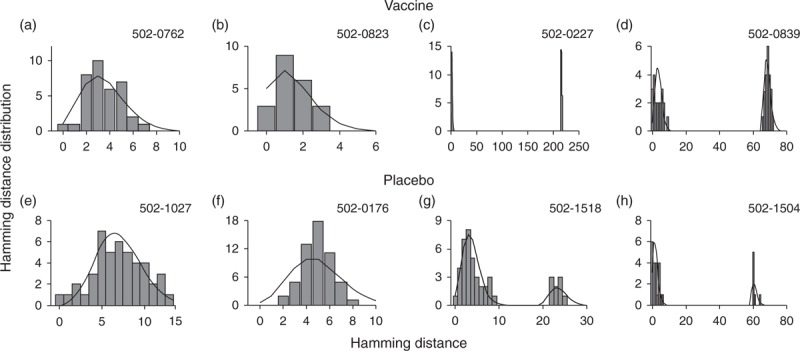
Fit of the shifted Poisson mixture model to STEP patients’ Hamming distance distribution.

We used the SPMM in Love *et al.*[22] to classify early infections in 59 patients as either single or multivariant transmissions. The SPMM, software available at http://www.hayounlee.org, is a mathematical representation of HIV gene sequence differences at a given time after infection. The SPMM was fitted to the Hamming distance distribution of full envelope gene sequences of each STEP participant, estimating days after infection and the number of founder variants (see the ‘Methods’ section). Figure 1 shows example cases of single-strain infections and multiple-strain infections in the vaccine and placebo groups. Our analysis estimates that 78% of the vaccinees (29 of 37) and 91% of the placebo recipients (20 of 22) conform to the model with a single founder virus (*P* = 0.29 for treatment group differences). The SPMM's classification of single and multiple founder variants was consistent with classification based upon analyses of phylogenetic trees and sequence diversities, with the exception of five patients [11]. Table 1 displays the SPMM fits to 29 vaccine and 20 placebo patients with single founder infections. The SPMM fits to the patients with multiple founder viruses are summarized in Table SI. This stratification enabled us to assess early viral escapes only within the patients whose infections originated from a single founder virus, avoiding false positives from multivariant infections.

The intersequence Hamming distance distributions (grey bars) of HIV *env* gene sequences obtained from four vaccinees (502-0762, 502-0823, 502-0227, and 502-0839) and four placebo recipients (502-1027, 502-0176, 502-1518, and 502-1504) in the STEP HIV vaccine trial. The sequence data were obtained from Ref. [11]. (a) The best fit of the shifted Poisson mixture model (black curve) to patient 502-0762's pairwise Hamming distance distribution (grey bars). The shifted Poisson mixture model estimated the number of founder variants as one and the duration of infection as 62.2 (40.4–84.1) days (goodness of fit *P* = 0.43). (b) The pairwise Hamming distance distribution of vaccine-treated patient 502-0823 along with the best fit of the shifted Poisson mixture model. The model estimated the number of founder strains as one and the duration of infection as 25.8 (9.8–41.8) days (*P* = 0.91). (c) The pairwise Hamming distance distribution from patient 502-0227 with the best fit of the shifted Poisson mixture model. The model estimated the number of founder variants as two and the duration of infection as 13.0 (4.0–21.9) days (*P* = 0.44). (d) The pairwise Hamming distance distribution from patient 502-0839 with the best fit of the shifted Poisson mixture model. The estimated number of founder variants was two and the estimated time after infection was 65.1 (43.9–86.3) days (*P* = 0.18). (e) The best fit of the shifted Poisson mixture model to placebo-treated patient 502-1027's pairwise Hamming distance distribution (grey bars). The model estimated that patient 502-1027 was infected by a single variant with the infection duration of 125.1 (95.7–154.4) days (*P* = 0.18). (f) The pairwise Hamming distance distribution from patient 502-0176 with the best fit of the shifted Poisson mixture model. The model estimated a single founder with the time since infection of 88.1 (64.6–111.6) days (*P* = 0.18). (g) The best fit of the shifted Poisson mixture model to the pairwise Hamming distance distribution of patient 502-1518's HIV envelope gene sequences. The estimated number of founder variants was two and the estimated time after infection was 65.8 (44.5–87.1) days (*P* = 0.05). (h) The pairwise Hamming distance distribution from placebo-treated patient 502-1504 with the best fit of the shifted Poisson mixture model. Two founder variants were estimated by the shifted Poisson mixture model, and estimated time after infection was 33.9 (17.0–50.8) days (*P* = 0.04).

We conducted a systematic analysis to identify early viral escapes that are enhanced by vaccination. We first constructed the library of CTL epitopes spanning the HIV Gag, Pol, and Nef proteins of the STEP vaccine construct, MRKAd5 [7,8]. The library consists of known HIV epitopes from the Los Alamos Laboratory HIV database and epitopes predicted by the software NetMHC [28,29] (see the ‘Methods’ section). The diversity of each epitope was then compared between the vaccinated and placebo individuals. We could have performed conventional hypothesis tests to identify any meaningful differences in epitope diversity between the two groups. Caveats of applying these tests would be that the vaccinated patients infected with a single strain  
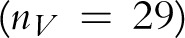
 outnumber their placebo counterparts  
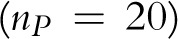
 given the relatively small sample size. Therefore, we conducted a permutation test in which we randomly permuted patient treatment groups to empirically assess the validity of the asymptotic *P* values. As detailed in the ‘Methods’ section, the empiric *P* value was obtained for each epitope through a total of 10 000 permutations between the vaccine and placebo groups; based on this empiric *P* value, the epitopes with significant differences were identified.

Out of the 2648 Gag, 5995 Pol, and 1345 Nef overlapping peptides screened, the diversity of four epitopes showed meaningful differences (at empiric *P* < 0.10) between the vaccine and placebo groups (Table 2). The identified epitopes shared several notable characteristics. First, all these epitopes were more diverse in the vaccine group than the placebo group. Second, all of the epitopes were located in Nef. This Nef specificity is notable given our observation that diversity in all Gag and Pol epitopes was consistently similar between the vaccine and placebo patients with a single-strain infection.

**Fig. 2:**
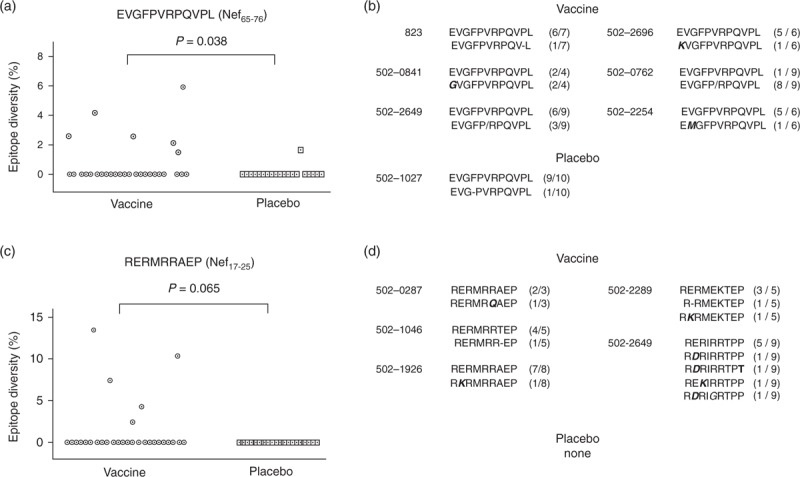
Differences in viral escape pattern between STEP vaccinees and placebo recipients.

The immunodominant Nef_65–76_ epitope, EVGFPVRPQVPL, showed significantly greater average epitope diversity within the STEP vaccinees who were infected by a single strain than within single-strain-infected placebo patients (*P* = 0.0038, permutation *t* test). The Nef_65–76_ epitope sequence was completely conserved within all placebo recipients except patient 502-1027, but six of 29 vaccinated patients showed diverse mutant variants (Fig. 2a and b). Interestingly, this Nef epitope has been reported to be located in the most frequently targeted region across ethnicities (34.1% in white to 52.5% in African-American) [31–33]. In addition, it was reported that vaccinated STEP study participants mounted autologous CD8^+^ T-cell responses targeting this peptide [34]. Taken together, greater diversity of this immunodominant epitope within the vaccine group suggests that viral escape was enhanced by the STEP vaccination.

(a) The diversity of epitope EVGFPVRPQVPL (Nef _65–76_) of 29 vaccinees and 20 placebo recipients whose infection was estimated to originate from a single founder virus. This epitope region was completely conserved in all but one placebo recipient, and six of 29 vaccinees showed nonzero epitope diversity. The epitope diversity differed significantly between the vaccine and placebo groups (*P* = 0.038), suggesting that viral escape is enhanced by vaccination. (b) Amino acid sequence variants of the Nef_65–74_ epitope in six vaccinees and one placebo patient that showed the signature of viral escape. In vaccinated patient 502-2649, the nine epitope sequences consisted of six wild-type epitopes (contained in the STEP vaccine) and three mutant peptides of the sequence EVGFP***I***RPQV. (c) The diversity of epitope RERMRRAEP (Nef_17–25_) of 29 vaccinees and 20 placebo recipients whose infection originated from a single transmitted virus. This region of the epitope was completely conserved in all 20 placebo recipients, and five of 29 vaccinee showed nonzero epitope diversity, implying that viral escape might have been enhanced by vaccination (*P* = 0.065). (d) Amino acid sequences of the Nef_17–25_ epitope in five vaccinees that showed variation as a signature of viral escape. The nine epitope sequences of vaccinated patient 502-2649 consisted of five different peptides – RERIRRTPP, R***D***RIRRTPP, R***D***RIRRTP***T***, RE***K***IRRTPP, and R***D***RI***G***RTPP. Within each of 20 placebo recipients, this epitope's sequences were identical.

Three other Nef CD8^+^ T-cell epitopes, RERMRRAEP (Nef_17–25_), HPMSQHGIE (Nef_166–174_), and EDPEKEVLEWR (Nef_174–184_) showed a marginally significant difference in the epitope diversity between the vaccine and placebo groups (Table 2). Although the RERMRRAEP epitope region was completely conserved within all the placebo-treated patients, five vaccine-treated patients showed the escape signature (Fig. 2c). Various epitope forms including R***K***RMRRAEP and RERMR***Q***AEP were observed in the five vaccine-treated patients, as shown in Fig. 2d. This epitope was previously confirmed to elicit IFN-γ responses [35–37] and overlapped with the reactive peptides to autologous CD8^+^ T cells of STEP vaccinees [34]. The epitope, HPMSQHGIE (Nef_166–174_), was reported to be recognized by CTL responses in acutely infected patients [38]. Supplementary Tables SII and SIII, showed all amino acid sequences of the four identified Nef epitopes within the 29 vaccine and 20 placebo patients, respectively.

As expected, the identified escape patterns showed a high level of association with each vaccine and placebo patient's HLA alleles. For instance, patients 502-0762, 502-0762, 502-2254, and 502-2649 in the vaccine group commonly have the allele HLA-A^∗^020 and showed viral escape in the Nef_65–76_ epitope with the segment, VGFPVRPQV, reported to be restricted by HLA-A^∗^0201 [39]. Around 67% of the escape events within the four Nef epitopes displayed consistent HLA restrictions with each patient's HLA alleles. HLA alleles of the escape patients were largely in agreement with those of patients who previously showed reactivity to the escape epitope. Previous studies reported that patients with HLA-A^∗^6801, A^∗^31, CW^∗^0401, or CW^∗^07 showed reactivity to peptides that overlap with the Nef_17–25_ epitope [12]. We observed that five STEP vaccine patients showed escape in this epitope, and all had one or more of these four alleles. Likewise, the other two Nef epitopes, Nef_65–76_ and Nef_174–184_, showed the same trend. In the Nef_166–174_ epitope, six vaccine and one placebo patients showed viral escape, but only two vaccine patients’ HLA alleles were consistent with either experimentally determined HLA restrictions or those of patients who showed reactivity to this epitope. Notably, all the four Nef epitopes showed a significant overlap with previously reported epitope hotspots that were specifically targeted by the STEP vaccination [34,40].

**Fig. 3:**
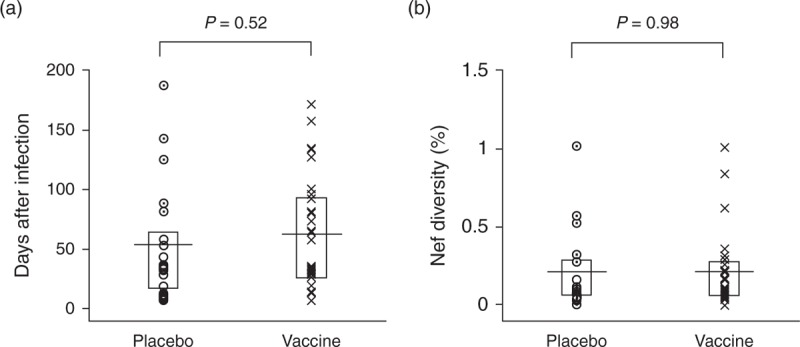
Infection duration and overall *nef* gene diversity of STEP vaccine and placebo patients.

The enhanced viral escape signatures observed in the vaccinees could be the result of longer infection times. The single-strain vaccine group's infection durations, estimated by the SPMM, were on average slightly greater than those of the single placebo group (62.0 vs. 52.9 days after infection); the difference, however, was not statistically significant [*P* = 0.52, analysis of variance (ANOVA)], as shown in Fig. 3a. We then examined whether the greater Nef epitope diversity in the vaccine group was representative of the entire Nef region. The overall *nef* gene diversity of vaccinated individuals with single-strain infections was comparable with that of the corresponding placebo patients (0.212 vs. 0.210%, *P* = 0.98, Fig. 3b). Although this overall diversity is comparable, greater epitope diversity in the single-strain vaccine group indicates that mutations preferentially occurred in the four Nef epitopes we identified. In addition, even when the phylogenetic classification in Rolland *et al.*[11] was used to separate the single-strain and multiple-strain infections, all the four Nef epitopes in Table 2 remained significant epitopes showing greater diversity in the single-strain vaccine. Collectively, these results suggest that the increased viral escapes were likely mediated by vaccine-induced T-cell responses.

(a) The infection duration estimated by the shifted Poisson mixture model for 29 STEP vaccinees and 20 placebo patients with a single-strain infection. The horizontal line denotes the mean of each group and the box plots the first and third quartiles. Infection duration did not differ significantly between the single-strain vaccine and placebo groups (ANOVA, *P* = 0.52). (b) *Nef* gene diversity comparison between the 29 vaccine and 20 placebo patients. The overall *nef* gene sequence diversity was comparable among the two groups (ANOVA, *P* = 0.98).

## Discussion

We discerned a robust signature of vaccine-enhanced viral escapes within HIV-1 Nef from the STEP trial. We compared the epitope diversity between vaccinated patients and plasma recipients whose infections originated from a single founder. In this way, we attempted to avoid misinterpreting high epitope diversity found in multiple-variant infections as a signature of viral escape. The difference in the level of epitope sequence variation within the vaccine-treated and placebo-treated individuals was statistically significant within the epitope EVGFPVRPQVPL (Nef_65–76_). Likewise, the diversity of the three other Nef epitopes showed marginally significant differences between the single-strain vaccine and placebo groups. We showed that the greater level of Nef epitope diversity was not due to any difference in infection duration. We also observed a high level of association between the identified escape events and each individual's HLA alleles. Furthermore, we observed that greater Nef epitope diversity in the single-strain vaccine group was not a result of greater overall *nef* gene diversity, collectively indicating the signature of vaccine-mediated viral escape.

The STEP vaccine-enhanced viral escapes observed in Nef reflect prior observations that initial CD8^+^ T-cell responses mainly target Nef. Numerous studies reported that the first wave of T-cell responses as early as 3 weeks after infection were Nef specific [12,24,41–44]. We speculate that vaccination primarily affects the first CD8^+^ T cells and thus specifically modulates viral escapes in Nef. Our observation is also analogous to a recent report that the emergence of variants of a Nef epitope was accelerated by narrowly focused CD8^+^ T cells primed by SIV vaccination [45].

The vaccine-enhanced escape patterns in Nef are largely in agreement with documented early escapes from the initial T cells targeting transmitted/founder viruses. Early escape in Nef_17–25_ from the first T-cell responses was reported in a serially followed acute patient [12]. The peptide EDPEKEVLEWR (Nef_174–185_) was previously noted as an early escape epitope with high entropy, indicating that the fitness cost of escape in this epitope is not high [46]. Strong CTL responses targeting the epitope, HPMSQHGIE (Nef_166–174_), were detected in acutely infected patients [38]. The epitope EVGFPVRPQVPL (Nef_65–76_) showed enhanced early escape in the STEP vaccine group. However, this epitope was previously classified as a late escape one as it showed mutations after 1 year [46], whereas an overlapping peptide_,_ QVPVRPMTYKAALDLSHF (Nef_73–90_) showed an acute escape signature [12]. Collectively, T-cell responses targeting the four epitopes identified in this study have been previously detected in acutely infected patients, suggesting that the STEP vaccination likely modulated the first T-cell responses and enhanced viral escape.

Detailed sieve analyses compared the genomes of viruses infecting the vaccine and placebo recipients of recent trials and revealed various vaccine-mediated genetic features [11,40,47]. A previous study of genome comparison between the STEP vaccine and placebo patients identified signature sites in Gag based on differences in the epitope divergence from the vaccine [11]. On the other hand, we found that escape signature sites distinguishing the vaccine from placebo patients were located in Nef. This regional difference originates from the following factors: we compared epitope diversity, not epitope divergence, to identify escape signatures and we excluded STEP infections with multiple transmitted/founder variants to remove false positive signals of nonzero epitope diversity. The genetic correlates for vaccine efficacy can be further depicted by examining HIV-1 genomes from multiple angles.

Characterizing how vaccines impact early HIV-1 evolution and viral escape is crucial for informing next-generation HIV-1 vaccines. Our observations suggest that virus escape is not suppressed by immunization with the entire gag/pol/nef HIV inserts. More recent vaccine designs focus on careful selection of vaccine composites to properly shape the T-cell landscape. From recent HIV-1 and SIV vaccine studies and other infections including malaria [48] and HCV [49], qualities of protective T-cell responses were shown to target only the most conserved subprotein domains [50–52], mount broad responses for multiple epitopes [5,49,53], and induce a high frequency of T-cell responses [48].

The STEP vaccination modulated the first wave of T cells targeting Nef, indicating that Nef-specific T cells were sensitive to vaccination. Thus we need to develop novel strategies that can induce protective Nef-specific T-cell responses. One direction would be to immunize with mutant forms of immunodominant Nef epitopes rather than with the most prevalent form of viruses to mount broad T-cell responses and thereby to suppress viral escape [54]. Our finding of vaccine-enhanced escape in Nef can guide better strategies for vaccine-primed first immune control.

## Acknowledgements

We thank Dr James Mullins (supported by P30AI027757) for providing the published sequence data from the STEP trial and helpful comments. We thank Jason Kaufman and Lucy Reynell for editing the manuscript.

Authors’ contributions: S.Y.P., W.J.M., and H.Y.L. performed mathematical and statistical analyses on the sequence data from the STEP trial and wrote the manuscript. All authors approved the final manuscript.

Financial support: This work was supported by NIH grants R01 AI083115 and AI095066 (to H.Y.L.).

### Conflicts of interest

There are no conflicts of interest.

## Supplementary Material

Supplemental Digital Content

## Figures and Tables

**Table 1:** Shifted Poisson mixture model estimates on 49 STEP patients with single founder infections.

Patient	Estimated days after infection	Goodness of fit *P* value^c^	Number of *env* gene sequences	Patient	Estimated days after infection	Goodness of fit *P* value^c^	Number of *env* gene sequences
Vaccine – single (*n* = 29)				Placebo – single (*n* = 20)			
502-0062	31.0 (12.1–49.8)	0.52	6	502-0053	33.6 (18.3–48.9)	0.14	10
502-0287	63.3 (30.2–94.5)	0.49	4	502-0176	88.1 (64.6–111.6)	0.18	11
502-0309	7.2 (2.8–17.1)	0.84	5	502-0322	36.2 (13.8–58.6)	0.47	5
502-0341	18.7 (5.2–32.3)	0.17	7	502-0346	32.2 (16.4–47.9)	0.39	9
502-0524	32.7 (17.6–47.9)	0.08	10	502-0364	57.8 (29.5–86.1)	0.04	5
502-0648	29.9 (7.2–52.6)	0.68	4	502-0388	28.3 (13.5–43.2)	0.55	9
502-0762	62.2 (40.4–84.1)	0.43	9	502-0525	14.6 (0.3–29.0)	0.55	5
502-0823	25.8 (9.8–41.8)	0.91	7	502-0572	7.2 (2.8–17.1)	0.84	5
502-0841	126.5 (79.6–173.4)	0.17	4	502-0717	18.1 (3.6–32.5)	0.59	6
502-0879	79.0 (46.0–112.0)	0.27	5	502-0923	13.4 (2.7–24.2)	0.49	8
502-0897^d^	81.1 (57.5–104.6)	0.40	10	502-0938^b^	82.5 (57.4–107.5)	0.13	9
502-1046	14.7 (0.3–29.1)	0.55	5	502-1027^d^	125.1 (95.7–154.4)	0.18	10
502-1055	12.0 (0.2–23.8)	0.62	6	502-1047	53.7 (26.5–80.9)	0.16	5
502-1191	157.4 (110.9–203.9)	0.60	5	502-1478^a^	28.0 (14.3–41.7)	0.60	5
502-1211	35.5 (13.5–57.5)	0.47	5	502-1799	10.9 (1.4–23.1)	0.36	5
502-1400	57.2 (29.2–85.3)	0.49	5	502-2495	12.0 (4.6–28.7)	0.73	3
502-1500	92.4 (52.6–132.2)	0.29	4	502-2586^d^	187.8 (141.1–234.6)	<0.001	6
502-1512	133.0 (103.7–162.4)	0.01	11	502-2622	143.2 (98.8–187.6)	0.66	5
502-1897^a^	13.1 (3.7–22.5)	0.15	5	502-2667	42.5 (18.5–66.6)	0.43	5
502-1926^b^	12.1 (0.2–23.9)	0.62	6	502-2794	42.9 (18.6–67.1)	<0.001	5
502-2136	28.5 (8.8–48.3)	0.19	5				
502-2241	171.3 (138.3–204.3)	<0.0001	11				
502-2254	134.6 (95.2–174.0)	0.58	6				
502-2289	95.4 (58.7–132.1)	0.32	5				
502-2349	73.1 (37.3–108.9)	0.26	4				
502-2437	14.5 (4.4–24.5)	0.75	10				
502-2649^d^	100.5 (72.6–128.4)	0.05	9				
502-2696	30.0 (11.4–48.6)	0.16	6				
502-2717	65.1 (39.7–90.4)	<0.0001	7				

^a^The left half of the genome was used. For all others, envelope sequences were used.

^b^Hypermutation signatures were removed.

^c^Less than 0.05 implies statistically significant deviation from the shifted Poisson mixture model.

^d^Patients with multiple-strain infection from the phylogenetic method in Ref. [11].

**Table 2:** CD8^+^ T-cell epitopes showing significant difference (*P* < 0.10) in epitope diversity between STEP vaccine and placebo groups.

Epitope	Average epitope diversity in vaccinated patients (%)	Average epitope diversity in placebo patients (%)	*P* value
EVGFPVRPQVPL (Nef_65–76_)	0.67	0.083	0.038
RERMRRAEP (Nef_17–25_)	1.31	0	0.065
HPMSQHGIE (Nef_166–174_)	1.07	0.22	0.065
EDPEKEVLEWR (Nef_174–184_)	1.33	0.30	0.083
