# Supplementary material for: Enhancement of viral escape in HIV-1 Nef by STEP vaccination
Source: AIDS. 2016 Sep 28;30(16):2449–58. doi: 10.1097/QAD.0000000000001202 (PMC5051524; doi:10.1097/QAD.0000000000001202)
Supplement: Supplemental Digital Content [file aids-30-2449-s001.pdf]

Subject	Number of Founder Variants	Estimated Days Post Infection	Number of sequences	Hamming Distances among Founder Variants	Goodness of Fit P Value#	Recombinant Removed
**Vaccine-Multiple**						
502-0227	2	13.0 [4.0 - 21.9]	6, 5	215	0.44	None
502-0491$	3	10.5 [1.4 - 22.4]	3, 1, 1	301, 296, 11	0.85	None
502-0839‡	2	65.1 [43.9 - 86.3]	6, 4	65	0.18	RH01, RH05
502-0961*	2	22.0 [13.2 - 30.7]	9, 1	67	0.99	None
502-1174*‡	3	34.7 [22.6 – 46.8]	2, 3, 3	69, 23, 92	0.05	FL04, FL11
502-1399	4	66.2 [36.1 - 96.4]	2, 1, 1, 1	35, 29, 26, 21, 19, 10	0.06	None
502-1619	2	60.6 [40.3 - 81.0]	8, 2	10	0.02	None
502-2305‡	5	17.1 [7.6 – 26.7]	7, 2, 2, 1, 1	31, 30, 27, 24, 24, 21, 20, 18, 17, 16	0.15	RH02
**Placebo-Multiple**						
502-1504	2	33.9 [17.0 - 50.8]	7, 1	59	0.04	None
502-1518	2	65.8 [44.5 - 87.1]	9, 1	20	0.05	None

Table SI. Shifted Poisson Mixture model (SPMM) estimates on STEP subjects with multiple founder infections.

*The left half of the genome was used. For all others, envelope sequences were used.
‡ Recombinant sequences were removed
#Less than 0.05 implies statistically significant deviation from the SPMM.
$Subject with single strain infection from the phylogenetic method in reference 11 of the manuscript.

Subject	EVGFPVRPQVPL (Nef$_{65-76}$)	RERMRRAEP (Nef$_{17-25}$)	HPMSQHGIE (Nef$_{166-174}$)	EDPEKEVLEWR (Nef$_{174-184}$)
502-0062	EVGFPVRPQVPL (6 / 6)	RERMRRTRA (6 / 6)	HPISQHGMD (6 / 6)	DDPEKEVLMWK (6 / 6)
502-0287	EVGFPVRPQVPL (3 / 3)	RERMRRAEP (2 / 3)<br>RERMR***Q***AEP (1 / 3)	HPMSQHGMD (3 / 3)	DDPEKEVLQWK (3 / 3)
502-0309	EVGFPVKPQVPL (5 / 5)	RERMRRTRP (5 / 5)	HPLSQHGMD (5 / 5)	DDPEKEVLQWK (5 / 5)
502-0341	EVGFPVRPQVPL (7 / 7)	RERMRRAEP (7 / 7)	HPGSLHGMD (7 / 7)	DDPEKEVLVWK (2 / 7)<br>DDPE***R***EVLVWK (4 / 7)<br>DDPE***R***E***I***LVWK (1 / 7)
502-0524	EVGFPVRPQVPL (10 / 10)	RERIRRAAP (10 / 10)	HPLNTHGMD (10 / 10)	DDPEKEVLVWR (10 / 10)
502-0648	EVGFPVRPQVPL (4 / 4)	RERMRRTEP (4 / 4)	HPMSLHGMD (3 / 4)<br>HPMSLHGM***G*** (1 / 4)	DDPEREVLQWR (3 / 4)<br>***G***DPEREVLQWR (1 / 4)
502-0762	EVGFPVRPQVPL (1 / 9)<br>EVGFP***I***RPQVPL (8 /9)	REKMRRTGP (9 / 9)	HPMSLHGME (9 / 9)	EDPEKEVLQWQ (9 / 9)
502-0823	EVGFPVRPQVPL (6 / 7)<br>EVGFPVRPQV-L (1 / 7)	RERMRRTEP (7 / 7)	HPMSQHGMD (7 / 7)	DDPEKEVLQWK (7 / 7)
502-0841	EVGFPVRPQVPL (2 / 4)<br>***G***VGFPVRPQVPL (2 / 4)	RERMARAEP (4 / 4)	HPMSLHGME (1 / 4)<br>HPMSLHGM***D*** (3 / 4)	DDPEREVLQWR (1 / 4)<br>***E***DPEREVLQWR (1 / 4)<br>DDPE***G***EVLQWR (1 / 4)<br>DDP***DG***EVLQWR (1 / 4)
502-0879	EVGFPVKPQVPL (5 / 5)	RERMRRTRP (5 / 5)	HPLSQHGMD (5 / 5)	DDPEKEVLQWR (3 / 5)<br>DDPEKEVLQW***K*** (2 / 5)
502-0897	EVGFPVRPQVPL (10 / 10)	RERMRRARP (10 / 10)	HPMCQHGMD (3 / 10)<br>HP***I***CQHGMD (6 / 10)<br>***Q***P***I***CQHGMD (1 / 10)	DDPEREVLMWK (9 / 10)<br>DD***L***EREVLMWK (1 / 10)
502-1046	EVGFPVKPQVPL (5 / 5)	RERMRR***T***EP (4 / 5)<br>RERMRR-EP (1 / 5)	HPLSQHGID (5 / 5)	DDPEKEVLVWK (5 / 5)
502-1055	EVGFPVRPQVPL (6 / 6)	RERMRQTEP (6 / 6)	HPMSQHGID (6 / 6)	DDPEREVLQWK (6 / 6)
502-1191	EVGFPVRPQVPL (5 / 5)	RDRMRRT-P (5 / 5)	HPGSLHGME (4 / 5)<br>HPGSLHG***L***E (1 / 5)	EDPEKEVLVWR (5 / 5)
502-1211	EVGFPVRPQVPL (5 / 5)	RERMRRAEP (5 / 5)	HPLNQHGMD (5 / 5)	DDPEREVLMWK (5 / 5)
502-1400	EVGFPVRPQVPL (5 / 5)	RERMRQTRV (5 / 5)	HPMNQHGVD (5 / 5)	DDPEREVLQWK (5 / 5)
502-1500	EVGFPVRPQVPL (4 / 4)	RERMRRA-V (4 / 4)	HPISQHGMD (4 / 4)	DDPEREVLQWK (4 / 4)
502-1512	EVGFPVRPQVPL (11 /11)	RERMRRTEP (11 / 11)	HPMSLHGME (4 / 11)<br>HPMSLHGM***D*** (7 / 11)	DDTEKEVLVWK (7 /11)<br>***E***DTEKEVLVWK (4 / 11)
502-1897	EVGFPVRPQVPL (5 / 5)	RERMEQTEP (5 / 5)	HPLSQHGMD (5 / 5)	DDPEREVLMWK (5 / 5)
502-1926	EVGFPVRPQVPL (8 / 8)	RERMRRAEP (7 / 8)<br>R***K***RMRRAEP (1 / 8)	HPMSQHGIE (8 / 8)	EDPEREVLVWK (8 / 8)
502-2136	EVGFPVRPQVPL (5 / 5)	RERMRRAEP (5 / 5)	HPMSLHGMD (5 / 5)	DDPEKEVLQWK (5 / 5)
502-2241	EVGFPVRPQVPL (11 / 11)	RERMRRAEP (11 / 11)	HPMSLHGMD (11 / 11)	DDPEGEVLQWK (11 / 11)
502-2254	EVGFPVRPQVPL (5 / 6)<br>E***M***GFPVRPQVPL (1 / 6)	RERMEKTEP (6 / 6)	HPMSQHGVD (6 / 6)	DDSEREVLQWK (6 / 6)
502-2289	DVGFPVRPQVPL (5 / 5)	RERMEKTEP (3 / 5)<br>R-RMEKTEP (1 / 5)<br>R***K***RMEKTEP (1 / 5)	HPMNLHGMD (5 / 5)	DDPEGEVLQWR (5 / 5)
502-2349	EVGFPVRPQVPL (4 / 4)	RDRMRRAEP (4 / 4)	HPMSQHGMD (4 / 4)	DDPEKEVLMWK (4 / 4)
502-2437	EVGFPVRPQVPL (10 / 10)	RDRMKRAEP (10 / 10)	HPINQHGMD (10 / 10)	DDPEREVLMWK (10 / 10)
502-2649	EVGFPVRPQVPL (6 / 9)<br>EVGFP**I**RPQVPL (3 / 9)	RERIRRTPP (5 / 9)<br>R***D***RIRRTPP (1/ 9)<br>R***D***RIRRTP***T*** (1/ 9)<br>RE***K***IRRTPP (1 / 9)<br>R***D***RI***G***RTPP (1 / 9)	HPMNQHGID (8 / 9)<br>H***S***MNQHGID (1 / 9)	DDPEREVLVWK (9 / 9)
502-2696	EVGFPVRPQVPL (5 / 6)<br>***K***VGFPVRPQVPL (1 / 6)	REKMRQHPP (6 / 6)	HPICQHGME (6 / 6)	EDADRDVLVWR (6 / 6)
502-2717	EVGFPVRPQVPL (7 / 7)	RDRMRRAPA (7 / 7)	HPANQHGVD (7 / 7)	DDPEKEVLMWK (7 / 7)

Table SII. Four Nef epitope sequences of 29 STEP vaccinees whose infections were estimated to originate from single founder variant.

Subject	EVGFPVRPQVPL (Nef$_{65\text{-}76}$)	RERMRRAEP (Nef$_{17\text{-}25}$)	HPMSQHGIE (Nef$_{166\text{-}174}$)	EDPEKEVLEWR (Nef$_{174\text{-}184}$)
502-0053	EVGFPVKPQVPL (10 / 10)	RERMRRAEP (10 / 10)	HPASLHGMD (10 / 10)	DDPEREVLVWK (10 / 10)
502-0176	EVGFPVRPQVPL (11 / 11)	RERMKRAEP (11 / 11)	HPMSLHGMD (11 / 11)	DDTEREVLQWK (11 / 11)
502-0322	DVGFPVRPQVPL (5 / 5)	REKMKRTEP (5 / 5)	HPMSLHGME (5 / 5)	EDPEKEVLVWR (5 / 5)
502-0346	EVGFPVRPQVPL (9 / 9)	RERMRRAEP (9 / 9)	HPMSQHGMD (9 / 9)	DDPEKEVLMWK (9 / 9)
502-0364	EVGFPVRPQVPL (5 / 5)	RERMRRARP (5 / 5)	HPLSQHGMD (5 / 5)	DDPEKEVLVWK (5 / 5)
502-0388	EVGFPVKPQVPL (9 / 9)	RERMRRAEP (9 / 9)	HPMAQHGMD (9 / 9)	DDPEKEVLMWK (9 / 9)
502-0525	EVGFPVKPQVPL (5 / 5)	RDRMRRAEP (5 / 5)	HPMSQHGMD (5 / 5)	DDPEKEVLIWK (5 / 5)
502-0572	EVGFPVRPQVPL (5 / 5)	RERMERAEP (5 / 5)	HPMSLHGAE (5 / 5)	EDPEKEVLMWK (5 / 5)
502-0717	EVGFPVTPQVPV (6 / 6)	RERMERTEP (6 / 6)	HPMSLHGME (6 / 6)	EDTEGEVLQWR (5 / 6)<br>EDTEGEV-QWR (1 / 6)
502-0923	EVGFPVRPQVPL (8 / 8)	RERMRRTEP (8 / 8)	HPMSQHGMD (8 / 8)	DDPEKEVLEWR (8 / 8)
502-0938	EVGFPVKPQVPL (10 / 10)	RERMQRTEP (10 / 10)	HPVSLHGME (10 / 10)	EDPEGEVLMWK (10 / 10)
502-1027	EVGFPVRPQVPL (9 / 10)<br>EVG-PVRPQVPL (1 / 10)	RERIRRAEP (10 / 10)	HPICQHGMD (8 / 10)<br>HPIC-HGMD (1 / 10)<br>HPICQHG***T***D (1 / 10)	DDPEREVLVWR (10 / 10)
502-1047	EVGFPVRPQVPL (5 / 5)	RDRMRRAPA (5 / 5)	HPMSQHGMD (5 / 5)	DDPEKEVLMWK (5 / 5)
502-1478	EVGFPVTPQVPL (5 / 5)	RERMRRAEP (5 / 5)	HPMSQHGVD (5 / 5)	DDPEKEVLMWK (5 / 5)
502-1799	EVGFPVRPQVPL (5 / 5)	KEKMKRTDP (5 / 5)	HPMNQHGMD (5 / 5)	DDPEREVLVWK (5 / 5)
502-2495	EVGFPVRPQVPL (3 / 3)	RERMRRAEP (3 / 3)	HPVNLHGMD (3 / 3)	DDPEKEVLQWK (3 / 3)
502-2586	EVGFPVRPQVPL (6 / 6)	RERMRRAEP (6 / 6)	HPMSLHGME (6 / 6)	EDPEREVLQWK (5 / 6)<br>EDPE***G***EVLQWK (1 / 6)
502-2622	EVGFPVRPQVPL (5 / 5)	RKRMKQTEP (5 / 5)	HPISQHGMD (5 / 5)	DDPEREVLQWK (5 / 5)
502-2667	EVGFPVRPQVPL (5 / 5)	RERMRRTEP (5 / 5)	QPMSRHGMD (5 / 5)	DDPEKEVLVWK (5 / 5)
502-2794	DVGFPVRPQVPL (5 / 5)	RDRMRRAEP (5 / 5)	HPINQHGVD (5 / 5)	DDPEKEVLMWQ (5 / 5)

Table SIII. Four Nef epitope sequences of 20 STEP placebo-treated subjects whose infections were estimated to originate from single founder variant.
